# A Critical Reassessment of the Role of Mitochondria in Tumorigenesis

**DOI:** 10.1371/journal.pmed.0020296

**Published:** 2005-10-04

**Authors:** Antonio Salas, Yong-Gang Yao, Vincent Macaulay, Ana Vega, Ángel Carracedo, Hans-Jürgen Bandelt

**Affiliations:** **1**Unidade de Xenética, Instituto de Medicina Legal, Facultade de Medicina, Universidad de Santiago de Compostela, Galicia, Spain,; **2**Centro Nacional de Genotipado (CeGen), Hospital Clínico Universitario, Santiago de Compostela, Galicia, Spain,; **3**Key Laboratory of Cellular and Molecular Evolution, Kunming Institute of Zoology, Chinese Academy of Sciences, Kunming, Yunnan, China,; **4**Department of Statistics, University of Glasgow, Glasgow, Scotland, United Kingdom,; **5**Fundación Pública Galega de Medicina Xenómica (FPGMX), Hospital Clínico Universitario, Universidad de Santiago de Compostela, Galicia, Spain,; **6**Department of Mathematics, University of Hamburg, Hamburg, Germany; University of Newcastle upon TyneUnited Kingdom

## Abstract

**Background:**

Mitochondrial DNA (mtDNA) is being analyzed by an increasing number of laboratories in order to investigate its potential role as an active marker of tumorigenesis in various types of cancer. Here we question the conclusions drawn in most of these investigations, especially those published in high-rank cancer research journals, under the evidence that a significant number of these medical mtDNA studies are based on obviously flawed sequencing results.

**Methods and Findings:**

In our analyses, we take a phylogenetic approach and employ thorough database searches, which together have proven successful for detecting erroneous sequences in the fields of human population genetics and forensics. Apart from conceptual problems concerning the interpretation of mtDNA variation in tumorigenesis, in most cases, blocks of seemingly somatic mutations clearly point to contamination or sample mix-up and, therefore, have nothing to do with tumorigenesis.

**Conclusion:**

The role of mitochondria in tumorigenesis remains unclarified. Our findings of laboratory errors in many contributions would represent only the tip of the iceberg since most published studies do not provide the raw sequence data for inspection, thus hindering a posteriori evaluation of the results. There is no precedent for such a concatenation of errors and misconceptions affecting a whole subfield of medical research.

## Abstract 2

The role of mitochondria in tumorigenesis remains unclear; in this paper Salas and colleagues raise concerns over many published studies

## Introduction

For more than two decades human mitochondrial DNA (mtDNA) has widely been used as a versatile tool to investigate different genetic aspects such as the origin and migration patterns of human populations or criminal casework in the forensic field. Specific mutations in the mtDNA genome are also suggested to be responsible for human diseases such as Leber hereditary optic neuropathy, myoclonic epilepsy associated with ragged-red fiber disease, MELAS syndrome, deafness, and inherited adult-onset diabetes (see [1] for a recent review). In the past few years, the putative role of mtDNA in cancer has received special attention. While many studies seem to support an active role of mtDNA in tumorigenesis [2,3], there are many caveats, and the issue has been highly debated [4–6].

Owing to the multiple steps involved in mtDNA analysis, systematic errors in mtDNA sequences are often found in the anthropological and forensic literature [7–11]. Thus one should also expect to detect similar problems in clinical investigations. More than half of published mtDNA sequencing studies contain obvious errors, no matter in which journal the investigation is published [12]. The consequences of such errors can be more or less dramatic depending on the subject or particular case under study. In the forensic context, a single mistake can lead to the false exclusion of an individual as the source of the biological material left at a crime scene or to a mismatch in comparisons of an mtDNA profile with forensic databases. Systematic errors can also lead to biological dogmas such as the maternal inheritance of mtDNA being brought into question [13]. In an oncogenetic context, flawed sequence data can lead to a conclusion of false association between seemingly causal variants and tumor instability.

A phylogenetic approach to the analysis of mtDNA profiles (in which the sequences under consideration are compared with the current database of complete sequences that make up the global mtDNA phylogeny) has been shown to be useful for assessing the accuracy of mtDNA data [14]. In the clinical context, such an approach allows mtDNA sequences to be assigned to haplogroups alias monophyletic clades (that is, groups of all mtDNA sequences derived from a common ancestor), according to the haplogroup-specific mutations they harbor, and offers clues for pinpointing flaws. The mutational processes that lead to a cancer (or the mutations accumulating during cancer proliferation) could hardly reproduce by chance (mutation by mutation) the long evolutionary routes between distant mtDNA haplogroups. Therefore, when a tumor sample is apparently distinguished from the corresponding normal tissue sample by (nearly) all the mutations distinguishing two very different haplogroups, then the only conclusion is that one of the two samples was contaminated or exchanged by mistake.

## Methods

### MtDNA Databases for the Interpretation of Human Population Variation

The use of large worldwide databases is of great help for identifying matching sequences and confirming membership to specific haplogroups, as well as for obtaining information about their geographical distribution (phylogeography). In order to study mtDNA variation properly it is necessary to take the full body of published mtDNA studies into consideration. While MITOMAP (http://www.mitomap.org/) provides a useful (but incomplete) listing of single mutations that have appeared in the older medical literature, direct reference to population databases of complete or nearly complete mtDNA sequences allows the inference of mutations that have occurred on evolutionary pathways between reconstructed ancestral sequences. Thus, a snapshot of the global mtDNA phylogeny and some of its representatives in all continents is given by the complete sequences of Ingman et al. [15], although the accompanying diagrams are devoid of the information that a medical geneticist would need, namely, a reconstruction of the coding-region mutations along the estimated phylogeny. The data of Herrnstadt et al. [16,17], which comprise only the coding region, give additional information, with the emphasis on European mtDNA, while Kong et al. [18] data contain East Asian complete genomes. Coble et al. [19] provided a considerable number of new complete mtDNA genomes, which were preselected according to frequent control-region haplotypes found in Europe. Most recently, Palanichamy et al. [20] obtained 75 complete sequences from haplogroup N sampled in India, and Achilli et al. [21] published 62 complete mtDNAs, covering most of the basal variation of haplogroup H.

The deeper parts of the global mtDNA phylogeny are expressed through a system of nested haplogroups, which are encoded by strings of letters and numbers in alternation, following specific rules [22]. In what follows we make use of this haplogroup nomenclature [18,20] in order to reference the pertinent sections of the phylogeny.

## Results

### The Unsuitability of an “Allelic” Approach

Uniparental markers, such as mtDNA, were newcomers to the field of human genetics, where classical nuclear markers had been predominant. Consequently, the analysis of the new markers proceeded in the traditional way by treating the segregating nucleotides at each polymorphic position in the sequence as alleles and treating each position independently so that haplotypes were disrupted, and the strong association of certain mutations along the phylogeny was disregarded completely. The cumulative listing of mtDNA mutations observed in patients or controls [23,24] is therefore not only rather uninformative but often misleading.

The strategy followed by Nishikawa et al. [25] will serve as a paradigmatic example of what is conceptually inappropriate. These authors sequenced the entire mtDNA genome of two individuals with hepatocellular carcinoma (HCC) and a liver specimen from one control, as well as the D-loop region (only nucleotides 100–600) of another six controls and 69 HCC specimens of Japanese subjects. They used an arbitrary mtDNA complete genome deposited in GenBank (accession number J01415) as a reference sequence. This sequence differs from the revised Cambridge reference sequence (rCRS [26]) by four mutations: A4985G, C11335T, C14766T (constituting three of the 11 errors of the original Cambridge reference sequence [27]), and A750G; it is actually an artificial sequence phylogenetically related to haplogroup H. When comparing the mtDNA of the control liver specimen with the J01415 sequence, they found only three differences. Judging from the context in their paper, the three differences, however, must be A263G, 315+C, and T489C. Thus, all sequences were erroneously scored at the four positions 750, 4985, 11335, and 14766. The presence of the substitution T489C indicates that the control lineage belongs to one of the haplogroups M and J, very likely to the former because haplogroup J is virtually absent in Japanese, but this is at odds with the meager number of differences to the rCRS. Even more alarmingly, since all samples screened for region 100–600 were claimed to harbor those three mutations in the D-loop, we would then expect no single sequence from haplogroup N (which embraces the East Eurasian haplogroups A, B, F, etc.) in 78 individuals. This is very unlikely when we consider the haplogroup distribution pattern in large Japanese mtDNA datasets [28], which testify to more than 30% of haplogroup N sequences. It therefore seems that contamination with some haplogroup M sequence had affected the samples. Finally, the authors compared the mtDNAs of two cancerous tissue specimens with sequence J01415 and found as many as 67 and 77 mutations, respectively (half of them also present in the paired noncancerous tissue specimens of the two patients). The two cancerous tissues seem to share several mutations, especially in the region 11000–16000 of the mtDNA genome rather than with their matched noncancerous tissues (see their Figure 1), which again would make contamination or sample mix-up plausible as a cause for the incidence of seemingly somatic mtDNA mutations in HCCs. There is no consistent way to allocate the mutations that separate the rCRS from the root of haplogroup H (or R) or the mutations distinguishing M and R in their Figure 1 (see [25]). It seems that massive oversight of mutations on the one hand, and contamination on the other, have shaped the picture presented. Since the total data obtained by the authors are not reported mutation by mutation, the likely causes of the sequencing dilemma cannot be reconstructed more precisely.

**Figure 1:**
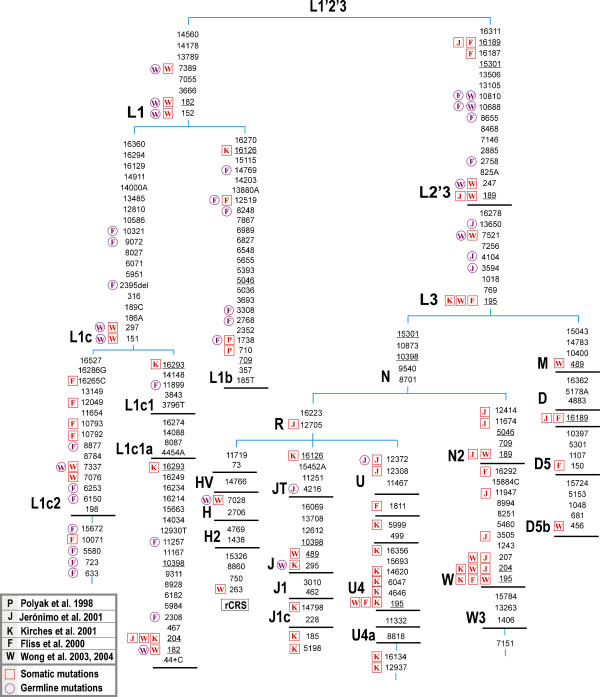
Portion of the Worldwide mtDNA Phylogeny That Explains the Most Relevant Contamination/Sample Mix-Up Episodes Erroneously Interpreted as mtDNA Instabilities in Several Kinds of Tumors or Detected as Germline Mutations, as Commented in the Text Capital letter-number codes designate haplogroups; bold bars indicate intermediate branching points as inferred from the total mtDNA phylogeny. Variation at position 16519, length polymorphisms of long C-stretches in HVS-I and II, and dinucleotide repeats at 522–523 are disregarded. All mutations are transitions except for those suffixed by A, G, C, or T (transversion) or del (deletion) or + (insertion of the specified nucleotide). Parallel mutations are underlined. Somatic (red squares) and germline (pink circles) mutations are indicated on the left side of each position in the tree as they appear in their original studies.

Yeh et al. [29] studied mtDNA in papillary thyroid carcinomas (PTCs). A whole paragraph of the discussion is dedicated to two (C7521A, A10398G) of the three missense mutations found in PTC cases. The authors failed to recognize that both C7521A and A10398G are familiar mutations in the mtDNA phylogeny. The latter is shared by nearly all haplotypes outside haplogroup N, whereas the former is common to virtually all members of the major African haplogroups L0, L1, and L2. It then seems that the authors' statement that “the 10398A>G and 7521G>A variants might not be totally innocuous…” (p. 2064) and that “one might speculate that these somatic mtDNA mutations are low penetrance modifiers of tumour risk…”(p. 2064) is most implausible, given the ubiquity of this mutational pair on the African continent. It is also remarkable that a meager number of 30 controls and nine fetal tissues without heteroplasmy at positions 7521 and 10398 led the authors to speculate that “this suggests that when these mutations are somatic, they are specific to PTCs (p. 2064).” As we shall see below, seemingly heteroplasmic or somatic mutations may be the result of contamination or sample mix-up and therefore would necessitate more sequencing and cloning efforts.

Yeh et al. [29] also followed a paradigm that is nearly ubiquitous in all those studies about the role of mtDNA and tumorigenesis, namely, the straightforward comparison of a patient group with an arbitrary control group, by counting mutations relative to the rCRS. A seemingly larger number of mutations in the patient group would normally reflect sampling effects in that different parts of the phylogeny are covered by the mtDNAs of patients compared to controls. In other words, controls and cases do not necessarily represent the same population and ethnic matching; this provokes a well-known effect in popular association studies that leads to spurious association between probands and the polymorphism/mutation under study. For example, only one (G15179A) out of 16 mutations that were found in PTC but not in controls and fetal tissues [29] is apparently a mutation not yet reported in normal mtDNA genomes from worldwide studies of the past 5 y. Worse, at least three PTC mtDNAs contribute more than one mutation to the list, which are inherited from the particular basal branch of the worldwide phylogeny the mtDNA belongs to. One haplogroup L1b lineage is responsible for mutations T710C and T3308C that are characteristic of this haplogroup as well as mutation T7389C specific to the superhaplogroup L1. Similarly, potential Native American mtDNAs from haplogroups D1 and B2 could have contributed two mutations each. The phylogenetic linkage of mutations therefore violates the tacit assumption of independence behind any claims of “significance.”

The most recent claim that one “can now add cancer to the list of mitochondrial diseases” (see [30], p. 724) in that mtDNA mutations are associated with a predisposition to prostate cancer should also be received with skepticism. The fact that a known pathogenic mutation such as T8993G can influence the rate of tumor growth cannot alone corroborate this claim—nor can a simple correlation study that contrasts cytochrome oxidase subunit I polymorphisms found in patients with those found in controls. For instance, mutation T6253C that is believed to be associated with prostate cancer [30] belongs to the characteristic mutations for the European haplogroup H15 [21] and both East Asian haplogroups D5 [18] and M13 [31]. To our knowledge it has not been reported yet that, in Japan where D5 and M13 thrive, a considerable number of men suffered from rapidly growing prostate cancer.

### Alleged Mutational Hotspots

Methodological procedures can be prone to sequence artifacts that can erroneously be interpreted as mtDNA mutational hotspots. Because of their nature, mutational hotspots emerge frequently in the mtDNA phylogeny and for this reason are well known in human population studies. A familiar example within the clinical literature is the unstable homopolymeric “C” track in the hypervariable segment II (HVS-II) region, from positions 303 to 309, but also positions 146, 150, and 152 [6] in the same segment. Turning it the other way around, rare or stable diagnostic variants are extremely unlikely to be mutational hotspots.

We highlight the unusual findings in Khrapko et al. [32] in this regard. After analyzing a short coding-region fragment of 100 bp by mutational spectrometry, these authors reached the conclusion that different human tissues and cells contained a remarkably similar set of hotspot point mutations. However, we observe that the hotspots they reported are correlated very poorly with the mutational spectra inferred from human populations. For instance, their two most important outstanding “hotspots” (positions 10068 and 10098 in their Figure 3) have never been detected in complete genome sequencing analysis (despite there being more than 1,700 complete mtDNA genomes available in the literature, covering most of the basal worldwide mtDNA phylogeny). To our knowledge, the remaining so-called hotspots have never been found in the human population literature either, with the exception of position 10084, which is associated with J1, K, and L1c lineages [16]. The real causes of this unexpected result remain obscure, since their methodology used to detect sequence variants (mutational spectrometry) is seldom used in the field, so one cannot exclude the possibility that it is strongly affected by artifacts.

The study by Reddy et al. [33] of patients with myelodysplastic syndromes also suggested the presence of novel mutational hotspots. Among them, positions 7264, 7594, and 7595 have never been detected in population studies, whereas others such as 7289 have been found in only a single sequence (*Homo sapiens* isolate T1–12 mitochondrion, complete genome [19]). Also unrealistic is the fact that 25 out of 52 mutational events listed in their Table III are transversions, whereas another 18 “instabilities” are indels; but only nine transitions (by far the most common type of change in mtDNA) were reported (one of these transitions [A7768G] is diagnostic for haplogroup U5b [20]). The transition:transversion ratio for their data contrasts significantly with very conservative estimates taken from hundreds of human population studies; additionally the extremely high prevalence of indels is certainly unrealistic. For similar and additional reasons, this study has been questioned by others [5]. Unexpected results must be corroborated with standard methodology and by independent studies; in this sense, the commitment of Reddy et al. [33] has not been published yet. In addition, we agree with Gattermann et al. [5] in that the detection of mutations within primer annealing sites is at least unorthodox. The explanations of Reddy et al. [33] are certainly not convincing and, of course, do not explain the most striking fact: why have these hotspots not been detected (even as private variants) anywhere else in population studies?

### Contamination and Sample Mix-Up

The recent work of Bandelt et al. [10] analyzed the causes and consequences of artificial recombinants, focusing attention on the forensic and population genetic literature. As predicted, mtDNA analysis in clinics does not escape the problem of contamination and sample mix-up. All the studies that we comment on below have a common denominator: contamination or sample mix-up of the tumor samples under study with exogenous mtDNAs. Typically, these findings usually lead to innocent erroneous interpretations and the concomitant development of a biological explanation or the invocation of a theory that would justify the role of such variants in tumorigenesis. A classical case constitutes the finding of three “somatic” homoplasmic mutations (T710C, T1738C, and T3308C) in colorectal tumor V478 [2]: these rather rare mutations all belong to the sequence motif for haplogroup L1b (see also [34]).

Fliss et al. [3] provide us with a pertinent example in the analysis of mtDNA sequences in tumor studies. Patient 884 (their Table 1; bladder cancer) shows a total of five mutations (T10071C, T10321C, A10792G, C10793T, and C12049T) all of which have been found in a haplogroup L1c2 lineage, namely, no. 173 in Herrnstadt et al. [16], which is related to the African lineage no. 48 in Ingman et al. [15]. Therefore, these mutations are extremely unlikely to have anything to do with tumorigenesis but rather represent an instance of contamination or sample mix-up involving a specific L1c2 mtDNA (Figure 1). Corroborating evidence comes from their Supplemental Table 1 (www.sciencemag.org/feature/data/1048413.shl, which lists the so-called new mtDNA polymorphisms detected at the time as being shared by matched cancerous and normal tissues. As many as 13 mutations recorded for the bladder cancer cases are also seen in lineage no. 173 of Herrnstadt et al. [16], including its seemingly private mutations A633G, A723G, T5580C, and T15672C. Not surprisingly, the five somatic mutations claimed to be somatic in Patient 884 were missed as polymorphisms in the bladder cancer patients. This strongly suggests that two amplicons of normal tissue from Patient 884 (one covering 10071–10793 and the other including 12049) were exchanged with (or contaminated by) tissues stemming from some other patient or patients.

The polymorphisms listed in Supplemental Table 1 of Fliss et al. [3] testify to further problems. Among the mutations that were found in lung cancer patients, four mutations point to haplogroup L1c and three additional ones (A2308G, C11257T, and T11899C) to a very specific branch of subhaplogroup L1c1a [15,35]. This being the case, the three mutations G2758A, C8655T, and A9072G from the evolutionary path between rCRS and haplogroup L1c should have been recorded there as well—but they were not. Neither had G2758A been reported for bladder cancer, although L1c was present there as well. The presence of another mtDNA haplogroup of African ancestry is documented in that table of polymorphisms, namely, the six mutations T1738C, A2768G, T3308C, A8248G, T12519C, and A14769G belong to the characteristic motif of haplogroup L1b. Five of them are associated with lung cancer, four with bladder cancer, and three with head and neck cancer patients. This means that a total of 1 + 2 + 3 = 6 mutations must have been missed in individual patients (as well as C8655T in the head and neck cancer case) since natural back mutations in such great number would be unrealistic. We conclude that sample contamination or massive oversight of mutations must have been the rule rather than the exception in Fliss et al. [3].

Jerónimo et al. [36] claim to have demonstrated the existence of specific patterns of somatic mtDNA mutations in prostate cancer. These authors also reported a spectacular case in the clinical literature: 18 somatic mutations detected in one patient (see their Table 1, patient 1). Surprisingly, many of these alterations conform to the familiar western European mtDNA haplogroup W [20]: A189G, T204C, G207A, A3505G, C11674T, A11947G, T12414C, and C12705T (Figure 1). In addition, their Table 1 suspiciously contains variants such as A3480G that identify haplogroup K, as well as A12308G and G12372A, which are characteristic of the larger haplogroup U, in which, haplogroup K is nested. Although A235G is a good candidate for haplogroup A, it too has been found within haplogroup K (USA.CAU.001306 in the SWGDAM database [37]), while other mutations such as T146C, A16183C (erroneously reported as A16183G in Jerónimo et al. [36]), and T16189C can be found on many haplogroup backgrounds (including haplogroup W). Such an unbelievable departure from random expectation represents a good candidate for cross-contamination between at least two different samples: one from haplogroup W and one from K. Therefore, there is no need to invoke the effect of endogenous factors or catastrophic mutagenic effects of exogenous exposure for this “hypermutated individual”: “Intriguingly, this patient worked for many years at a chemical plant” (see [36], p. 5196).

Similarly, Kirches et al. [38] carried out pairwise comparisons between glioma samples and adjacent brain tissues of 55 patients. Strikingly, patient 2 (their Table 1, p. 536) accumulated a total of 17 homoplasmic transitions, in contrast with the rest of the instabilities reported, which are all length variations of (di-)nucleotide repeats in the control region, except for one homoplasmic change between glioblastoma and normal tissue (namely, at position 72). The somatic mutations reported for patient 2 split into two mutational motifs with respect to rCRS: T195C, T4646C, T5999C, A6047G, A12937G, T13124C, C14620T, C16134T, A16293G, T16356C, and T16519C in the glioblastoma and G185A, T204C, C295T, A5198G, T16126C, and T14798C in the normal tissue (Figure 1). Here we assume that Kirches et al. [38] have misrecorded A12937G as A12936G and misassigned A5198G to the glioblastoma or interchanged the nucleotides A and G at 5198 by mistake. Our interpretation thus posits that 11 of the 13 gliostoma mutations confirm to the motif of a particular branch of haplogroup U4a [6,17,39], thus leaving only A16293G and T13124C as potential private mutations. All six normal tissue mutations point to the (yet unnamed) branch of haplogroup J1c defined by A5198G (compare with Coble et al. [19] and Herrnstadt et al. [16,17]; see Figure 1). Although the authors identify most of these substitutions as known polymorphisms in humans, they failed to recognize the most plausible justification for these results, namely, contamination or sample mix-up: “Whatever the mechanisms of mutation may be, the fast selection of certain tumor mitochondria, as demonstrated by Polyak et al. (1998), offers the only simple explanation for the accumulation of 17 homoplasmic mutations in a single tumor sample…” ([38], p. 537]). As usual, unfortunate results lead to the formulation of unjustifiable conclusions: “The occurrence of mutations in tumors at known polymorphic mtDNA sites and the dominance of transitions suggest a common mechanism generating germline mtDNA polymorphisms and somatic mutations” ([38], p. 536).

Wong et al. ([40], p. 3870) stated that “Kirches et al. (2001) reported 19 somatic mtDNA mutations found in a single glioblastoma. One of our [medulloblastoma] patients harbored 11 somatic mtDNA mutations.” Unfortunately, this study [40] is another example of an artifactual result: leaving aside a length polymorphism of a C-stretch (in HVS-II), the following 11 mutations are recorded in Wong et al.'s patient (their Table 1, case no. 124): C151T, C182T, G246A, A297G, G317A, G7337A, G7521A, G7337A, T7389C, T15904C, and A15937G. Three of these mutations have actually been shifted by one position and should read G247A, G316A, and T15905C instead. Then except for T15905C and A15937G these mutations can be found in the African haplogroup L1c2 (Figure 1) [15,35], and compare with lineages no. 173 and no. 328 of Herrnstadt et al. [16,17]). It is then probably no coincidence that the “novel germ-line variation” reported in their Table 1b ([40], p. 3869) also testifies to two mutations, G10688A and T10810C (previously listed by Fliss et al. [3]), that would be observed with all haplogroup L0 and L1 sequences. In a recent report from the same group [41], patient HE19 was found to harbor 10 differences between DNA in liver cancer and normal tissue. Six of them (A189G, C194T, T195C, T199C, T204C, and G207A) in the tumor tissue point to haplogroup W (see, for instance, USA.CAU.000453 in the SWGDAM database; Figure 1), whereas three mutations, C456T, T489C, and (523–524)del, in the normal tissue could point to the East Asian haplogroup D5b (compare with THA.ASN.000058 in the SWGDAM database).

The study carried out by Liu et al. [42] on ovarian carcinomas includes at least one instance suspicious of sample mix-up or contamination. Namely, the normal tissue of patient OV88 carries mutation A249del (with no mutation at 489) characteristic of haplogroup F, whereas the tumor mtDNA shows the mutations T146C, T199C, T489C (characteristic of haplogroup M7c [43,44] plus T152C (which has also been observed in M7c sequences from East Asia; for example, in CHN.ASN.000337, JPN.ASN.000103, and THA.ASN.000048 from the SWGDAM database). Since this array of homoplasmic HVS-II mutations matches a pathway in the Chinese mtDNA phylogeny, we are led to conclude that the ovarian cancer mtDNA and the serum and normal tissue mtDNA of OV88 likely came from two different individuals. In their Table 1, many mutations point to several East Asian haplogroups (see [18]), and thus these mutations do not have anything to do with ovarian carcinomas (see also Figure 1).

Chen et al. [45] aimed at tracing somatic mutations in 16 cases of prostate cancer, by sequencing the highly hypervariable mtDNA control region in subjects with prostate cancer. Their Table 1 reports a patient (case 1) bearing eight “instabilities,” namely, mutations A16182C, A16183C, T16189C, C16232A, T16249C, G16274A, T16304C, and T16311C, leaving aside (522–523)del. Except for G16274A, which seems to be a private mutation, this is unmistakably a HVS-I haplotype belonging to haplogroup F1b, which is frequently found in China [18,43,44]. Not accidentally, all these variants were detected as heteroplasmies; therefore, this represents a perfect instance of contamination from a biological source carrying this F1b haplotype. Table 1 testifies to yet another highly suspicious example: case 4 carries nine somatic near-homoplasmic mutations (besides a C-stretch length polymorphism), among which A73G, G499A, A16182C, A16183C, T16189C, and T16217C happen to constitute the control-region mutations (relative to rCRS) of a genuine member of the East Asian haplogroup B4b [18], except for position 263, at which the vast majority of mtDNA sequences agree anyway but differ from rCRS. Additional information about case 4 is given in Chen et al. [46], where the B4bd characteristic mutation G15535A is reported. Moreover, among several serial tumor sections, one (C_1_) confirms haplogroup status B4b with G499A. On the other hand, the mtDNA of section C_2_ is clearly a member of haplogroup K1a, whereas the other sections would be compatible with haplogroup HV status. Therefore, multiple sample mix-up or contamination events are the most plausible cause underlying the seemingly close relationship among cases 1, 4, and 6, which they have instead interpreted as follows: “The nonrandom distribution of somatic mutations raises the possibility that certain constellations of sequence variation might be prone to somatic mutations”([45], p. 6472). When Chen et al. ([45], p. 6471) claimed that “the somatic mutations cannot be explained by experimental error or by contamination of nuclear mtDNA pseudogenes,” this may have been so, but profuse sample mix-up or cross-contamination perfectly explains the results.

The recent study of mtDNA control-region mutations in patients with esophageal squamous cell carcinoma [47] constitutes another good case for sample crossover. The mtDNA of the normal esophageal tissue of case 21 bears mutations C150T, C16067T, A16164G, A16171G, C16172T, A16182C, A16183C, T16362C, and T16519C and therefore belongs to haplogroup D5a. Interestingly, the blood sample shared all mutations with the normal esophageal tissue, except for having heteroplasmies at positions 16067, 16164, and 16171. In contrast, the tumor mtDNA contains C16184T, T16298C, T16443C, G16470A, G16471A, G16473A, and T16519C, which point to haplogroup M8a. Since only the mutations were reported that distinguish tumor mtDNA from blood mtDNA, we can expect that both actually share the motif A73G, A263G, T489C, T16189C, and C16223T [18]. The contrast between the two different sequences is well reflected by the pair CHN.ASN.000113 and CHN.ASN.000270 in the SWGDAM database. It then seems that one mutation in the blood sample was overlooked (at position 16266) and one in the tumor sample (at position 16319), but this cannot disturb the clear-cut haplogroup allocations. Case 20 [47] testifies to yet another sample mix: here tumor and adjacent normal tissue bear the same mtDNA variants (except for one heteroplasmy at 16266) in HVS-I, namely, C16185T, C16223T, C16260T, and T16298C, whereas the mtDNA sequence from blood is reported to have C16256T, C16270T, and A16399G. We are thus seeing here the clean contrast between a haplogroup Z and a (West Eurasian) haplogroup U5a1 sequence.

## Discussion

Somatic mutations, in a heteroplasmic or a homoplasmic state, can occur in all kinds of tissues and body fluids of patients affected by cancer or genetic diseases and in healthy controls [4,48,49]. The problematic findings of Polyak et al. [2] and Fliss et al. [3] have been uncritically accepted [50] and cited in virtually every study of perceived mtDNA alterations in tumors. There seems to be a general expectation that the amount of somatic mutation can be elevated in tumors. The problem then is that instances reported with a whole array of seemingly somatic mutations would confirm this expectation and be taken at face value instead of as a hint at contamination or sample mix-up. The main consequence of such sequencing disasters is that most of these flawed results are not filtered out from the clinical literature, thus adding more noise to the interpretation of the role of mtDNA in the complex tumor process. This eventually leads to a vicious cycle of ill-based interpretations of mutational variation in tumors.

We have detected innumerable deficiencies in the clinical literature related to the analysis and interpretation of mtDNA data in tumor samples. There is no precedent that we know of in the genetics literature for such a high number of flawed papers (most of them published in high-rank journals), which affect a whole subfield of clinical research. Since what we show here is based on the extremely meager information generally available in these published reports, we have every reason to believe that this is only the tip of the iceberg. Note also that the database of coding-region variants in natural populations is still limited (although currently comprising more than 2,100 complete genomes), so some more mistakes in this literature await detection. Moreover, we must keep in mind that the phylogenetic approach used here is certainly not able to detect all errors.

We have found that the vast majority (>80%) of the studies dealing with potential functional implications of the mtDNA molecule in tumorigenesis (and providing data for inspection) are based on faulty data with surreal findings. The present report should lead us to reconsider the role of mtDNA in tumorigenesis. Probably we should abandon the exciting findings unleashed as a result of the many sequencing failures that accumulated during this last decade. A model consisting of basically two main stages [6]—namely, (i) accumulation of homoplasmic mutations in mtDNA-unstable sites during tumorigenesis, and (ii) a consequential effect on the cell physiology—is still valid in order to explain the mtDNA changes occurring during the tumoral process.

The clues to understanding the causes of pitfalls in mtDNA sequencing are extensively discussed [7–12,14,17]. Degraded DNA or extremely low quantities of DNA from old frozen samples or inadequately stored samples (in paraffin, for example) used in many clinical/oncogenetic studies would explain the notoriously low quality of sequence results as well as an elevated risk of contamination. For instance, one can only obtain very small amounts of DNA using the laser-capture microdissection technique employed by Chen et al. ([45]; see above) to retrieve cancerous and noncancerous samples from serial tissue sections. Contamination and sample degradation would greatly affect the quality of DNA during the subsequent processes and finally contribute to rich mtDNA heterogeneity in the sequence. In these situations of limited quantities of endogenous DNA, the clinical geneticist would do well to employ many of the checks for authenticity proposed for ancient DNA studies [51]). In a way, the current situation in the field of carcinogenesis and mtDNA resembles the state of the art of ancient DNA sequencing in those early days where loads of contaminated samples were amplified and claimed to yield “mummy mtDNA.”

It is unfortunate that clinical studies in oncogenetics do not routinely report comprehensive sequencing results. This has two important consequences (see also [6]): first, the phylogenetic interpretation of the spectra of the mtDNA variants found is limited, and second, the phylogenetic proofreading of sequence data cannot be carried out properly by referees and readers.

In short, we advise authors and editors of scientific journals that (i) special care must be taken for sequencing and documentation since conclusions fully depend on the sequencing data; (ii) raw sequence data must be made fully accessible to referees and readers in order to allow a critical evaluation of the results [11,12] and proofreading during the reviewing process. Although this sounds routine, it is striking to observe that in the clinical literature related to tumorigenesis, we have not seen cases—with a very few exceptions (for example, [6])—that provide the complete primary sequencing results; (iii) referring to the mutation lists in MITOMAP is not sufficient; in addition, the complete record of the data from the population genetics field should be consulted as well.

The use of phylogenetic tools is highly recommended for the medical field, not only for the purpose of data analysis but also in the design of appropriate mtDNA studies [52]. In this way, the distinction between neutral polymorphisms in human populations and the mutations associated with the tumor process [6] or with other human disorders [53,54] stands a chance of being realized.

## Supporting Information

### Accession Numbers

The GenBank (http://www.ncbi.nlm.nih.gov/Genbank) accession number for *Homo sapiens* isolate T1–12 mitochondrion complete genome is AY495278.1.

**Patient Summary**

**Background**

DNA carries the genetic “blueprint” of all living things, and everyone has slightly different DNA from everyone else. DNA is found in the cell nucleus but also in other parts (organelles) of the cell called mitochondria. Techniques have been developed to analyze the differences between the mitochondrial DNA (mtDNA) of different people. Several scientific and medical uses are now being made of these tests, and the results have been stored on international databases.

**Why Was This Study Done?**

Many scientists believe that faults (mutations) in mtDNA play a part in the development of cancer and that tests that look for faulty mtDNA are a way of diagnosing cancer in its very earliest stages. (This could be important because cancer treatment is more effective if done early.) Other scientists claim that there have been experimental errors in the study of mtDNA and cancer (for example, samples may have been mixed up, or one sample may have been contaminated with cells from another) and that faulty mtDNA is not a sign of cancer. These authors wanted to investigate whether the previously published studies were correct.

**What Did the Researchers Do and Find?**

The authors of this paper point out that, if the mtDNA from a cancer tumor and the mtDNA of normal tissue from the same patient are shown by analysis to differ not just a little, but considerably, then this cannot be the result of a fault appearing in the tumor mtDNA. Instead it must mean that a mistake was made in the laboratory by mixing up DNA from different human sources. The researchers looked at international databases of mtDNA and the mtDNA phylogeny (evolutionary reconstruction of the human mtDNA lineages), and their conclusion was that most of the differences between tumor mtDNA and normal mtDNA are the result of experimental error. According to the authors, the experimental process routinely followed in these studies favors this kind of error.

**What Do These Finings Mean?**

The widely held view of scientists about the role of mtDNA in the development of cancer appears to be wrong. We do not know what role, if any, mtDNA plays.

**Where Can I Get More Information Online?**

This is a very specialized paper with no immediate implications for change in the way in which cancer is treated. Here are some sources of general information on cancer.

National Cancer Institute (US):

http://www.cancer.gov/

Cancer Research UK:

http://www.cancerresearchuk.org/aboutcancer/?version=1/

## Abbreviations

HCC: hepatocellular carcinoma
HVS: hypervariable segment
mtDNA: mitochondrial DNA
PTC: papillary thyroid carcinoma
rCRS: revised Cambridge reference sequence
